# Opportunities for nurses to address employee voice in health care providers: a scoping review

**DOI:** 10.1186/s12912-024-02331-y

**Published:** 2024-09-13

**Authors:** A. Kepplinger, A. Braun, A. Fringer, M. Roes

**Affiliations:** 1 Department of Nursing Science, Faculty of Health, Witten/Herdecke University, Witten, Germany; 2 Department of Health Sciences, Institute Nursing Science, IMC University of Applied Sciences Krems, Krems, Austria; 3 Institute Health Management, IMC University of Applied Sciences Krems, Krems, Austria; 4 Institute for Management and Economics in Healthcare, UMIT Tyrole, Hall, Austria; 5 Institute of Nursing, School of Health Sciences, Zurich University of Applied Sciences ZHAW, Winterthur, Switzerland; 6 Deutsches Zentrum Für Neurodegenerative Erkrankungen (DZNE), Witten, Germany

**Keywords:** Employee voice, Speaking up, Employee engagement, Opportunity, Health care provider, Nursing staff

## Abstract

**Background:**

Employees’ decision to speak up or to stay silent can have implications for health care providers, employees and people who need care. As a result, a shift is needed from blindly following guidelines to implementing a sustainable proactive organizational culture in which employees, especially nurses, can evaluate their work environment and take advantage of growth opportunities. The aim of this review is to analyse the characteristics of employee voice opportunities in the health care context, particularly for nurses.

**Methods:**

The search was conducted in April 2023 in the following databases: MEDLINE via PubMed, CINHAL via EBSCO, Scopus via Elsevier, Wiley/Web of Science and Cochrane Library. The search results were imported into the COVIDENCE program and screened by two researchers separately. We used the following search components: health care organization, opportunities, and employee voice. The review followed the PRISMA-ScR guidelines. We identified 951 studies in five databases and via citation tracking. After we removed 102 duplicates and screening 839 titles and abstracts, 23 full texts were assessed. According to our inclusion and exclusion criteria, we included 9 studies.

**Results:**

Three main characteristics of employee voice opportunities that need to be considered to enable nurses to have a voice in the organization were identified. These main categories are individual factors, organizational culture, and available voice channels. It is not possible to rank them in order of importance; they are interrelated.

**Conclusions:**

To conclude, employee voice is a process. In order for utilize employee voice opportunities, individual employee factors, organizational culture and its embedded context must be considered. Individual internal and external motivation, which is influenced by socio-cultural aspects and work hierarchies, must also be considered for successful use of opportunities.

**Supplementary Information:**

The online version contains supplementary material available at 10.1186/s12912-024-02331-y.

## Introduction

Employees’ decision to speak up or to stay silent can have implications for health care providers, employees and people who need care [1]. An important perspective on the topic of speaking up and silence is safety culture, which, as Lainidi et al. [2] note, makes up a major part of the existing literature. In considering the global shortage of nurses and the International Council of Nurses (ICN) clear call to invest in nurses' working conditions to improve health system effectiveness [3], it is recommended that voice and silent research should focus on the organizational improvement perspective as well as the employee perspective [2]. This recommendation is based on the understanding that voice and silence manifest differently in varying contexts. Specifically, security concerns and organizational development matters require distinct approaches [2]. Consequently, to promote high-quality person-centered care in a context with workforce challenges such as nursing turnover, as well as the different needs and requirements of different generations and populations, health care providers need to invest in creating a productive and safe work environment [4, 5]. This leads to the question what kind of opportunities could be identified to support the voice and silence of nurses to foster a productive and safe work environment.

### Background

If employees choose to *stay silent,* this indicates that they are aware of certain issues and fail to address them [6], often because they are afraid to share their perspective [7]. Consequently, important information or concerns about the organization are withheld, and mistakes can occur or further development becomes impossible [6–8]. The opposite of staying silent is speaking up. Speaking up means speaking out about meanings, concerns, and inquiries when there are uncertainties [1, 9–11]. There are a various definitions and approaches of speaking up and staying silent in the literature. Most definitions of speaking up emphasize the safe treatment of people in need of care, error management and whistleblowing, as well as on team communication [1, 2, 12]. However, speaking up and staying silent also involves an organizational perspective, having a voice in an organization as an employee [2, 13]. This engagement element of workplace democracy is known as employee voice. Employee voice refers to all organizational structures, mechanisms or practices in which employees participate and through which they try to influence their work and the performance of their organization [13, 14]. It is necessary to mention that the definitions come from different fields of organizational behaviour not only specific from the health care sector. Kolbe and Grande [1], Mannion et al. [12] and Lainidi et al. [2] focus specifically on voice and silence in healthcare organizations. While there is no clear definition of employee voice in health care [2], there are studies that attempt to analyze the various challenges of employee voice in health care. Challenges in the healthcare sector are generally related to a variety of social, hierarchical factors and the measurement of voice in health care. As collaboration in health care is multidisciplinary and employee voice affects all professional groups, studies have included different healthcare workers, but primarily nurses and physicians [1, 12, 15].

Nurses, in particular, have been shown to be reluctant to express their opinions and to have low self-efficacy regarding safety precautions [16]. For this reason, this review attempts to focus on nurses. Recent evidence from the pandemic addresses this issue and shows that opportunities to learn from nurses to optimize processes have been missed. It has been found that, especially in times of crisis where short-term turnarounds change patient safety and personal protective equipment every day, it is necessary that nurses have a voice [17]. According to Umoren et al. [18], one of the barriers that explains why health care professionals stay silent is the hierarchical culture in health care organizations. Other influencing factors on voice and silence include organizational identification [19] and trust from the management [20]. The consequences of employees staying silent are well researched [8, 21–23]. For example, if an employee does not feel valued, they perceive a lack of control or experience employee cognitive dissonance, which is regarded as low commitment, low trust, and further low internal motivation; this can result in low satisfaction, turnover, stress, and withdrawal [8]. Holland et al. [21] found that mechanisms for employee voice and responsive approaches to addressing the concerns of nurses are important for lowering burnout and promoting employee well-being. Lee et al. [24] identified factors that facilitate or inhibit nurses’ willingness to raise voice regarding patient safety in East Asia. The review results indicate that various factors, including socio-cultural, organizational and team-related, as well as individual factors, can either encourage or discourage individuals to raise their voices or remain silent. Another study by Lee et al. [25] shows that positive factors to promote the voice of staff include a culture of safety, management support, positive role models, familiarity with others, and personal characteristics. Negative factors include hierarchies, power differences, seniority, length of service, relationship anxiety and heavy workloads [25].

In their review, Lainidi et al. [2] summarize influencing factors on voice and silence and developed a framework. The *framework of conditions and issues that affect employee voice and silence*, describes three aspects of silence and voice: motivation, behaviour and outcome. The conditions that influence the three aspects of voice and silence are organizational, leadership, team, and individual. Additionally, the type, orientation, context, and severity of the problem also influence these three aspects of voice and silence. A fundamental aspect of supporting employee voice is the comprehension of the motivating factors [2].

In summary, individual, organizational and contextual characteristics influence nurses' decisions about whether to raise concerns and initiate change or remain silent. Therefore, an organizational culture is needed that encourages nurses to raise their voice and address negative factors. Managers can create an environment that supports nurses by using transformational leadership and developing strategies to promote nurses' perceived influence and psychological safety, by inviting and valuing staff, creating an open communication culture, giving feedback and by helping to understand the importance of all nurses' voices. And, by developing different strategies as the influences on voice and silence are different [26–29]. A review of interventions to promote speaking up in health care was conducted, encompassing all speak-up initiatives and focusing on workplace strategies. Their focus was directed towards the factors that needed to be considered in the implementation phase. Their results showed, that existing research as well national and international policy contexts are rarely considered and built upon when developing speak-up interventions. Furthermore, raise your voice is shaped by socio-cultural norms and values outside the organization [30]. This review is distinct from previous ones in that it concentrates on nursing and the characteristics of opportunities, as well as considering the various definitions of employee voice.

## Aim

The review aimed to identify opportunities that address employee voice behaviour of nurses and describe their characteristics. To develop the search components, the question is integrated into the PICo (population, phenomenon of interest, and context) [31], as shown in Table 1.

**Table 1:** PiCo schema

P:	Population	Nurses
I:	Phenomenon of interest	Opportunities
Co:	Context	Employee voice

## Methods

To achieve this aim, we conducted a scoping review. The scoping review was chosen because of the broad topic of employee voice, its many definitions, and the lack of empirical evidence on opportunities for nurses addressing employee voice behavior in health care organizations. Furthermore, it is not a question of analyzing the effectiveness of the various mechanisms of employee voice, but rather of identifying the characteristics of each [32, 33]. To conduct the scoping review, the research team followed the recommended steps for scoping reviews by the Joanna Briggs Institute [34]. The steps recommended by the Joanna Briggs Institute [34] were used by KA to ensure transparency: (i) defining and aligning the objectives and the question.

The researchers were guided in the conduct of the review by the following search question:

Which opportunities for nurses are mentioned in the literature to address employee voice in health care organizations, and what are their characteristics? (ii) developing and aligning the inclusion criteria with the objectives and question, (iii) describing the planned approach to evidence searching, data extraction, and presentation of the evidence. The developed plan was discussed by the research team and the search was carried out on this basis by KA. To support this search, the manual for literature searches in specialized databases by Nordhausen and Hirt [35] was also used and a search protocol was created by KA based on it. (iv) searching for the evidence, (v) selecting the evidence, (vi) extracting the evidence, (vii) analysing the evidence, (viii) presentation of the results, and (ix) summarizing the evidence and making conclusions [34]. The following chapters (3.1, 3.2, 3.3, 3.4 and 3.5) describe steps (ii)-(vii) in detail.

### Inclusion and exclusion criteria

We used the following inclusion and exclusion criteria (Table 2). The criteria are based on the aims of the review. It should be noted that only studies that actually focused on opportunities (such as interventions and tools) were included in terms of the context employee voice. Included studies have used for example a management tool to support employee voice and described the role of management in relation to the opportunity to address employee voice. Studies were excluded if their focus was only on leadership style and did not describe opportunities for employee voice. A further exclusion criterion was studies that did not include nurses.

**Table 2:** Inclusion and exclusion criteria

Criteria	Inclusion	Exclusion
Types of participants	Nurses, health care organization, health care provider, long-term care, aged-care facilities, hospitals	Other areas like economy, banking, the financial sector or manufacturing companies; only focus on other health care professionals like health care worker or physician
Concept	Employee voice	
Context	Opportunity, intervention, tool	Focus just on leadership style types
Language	No limitations	

### Search strategy

The search was conducted in April 2023 in the following databases: MEDLINE via PubMed, CINHAL via EBSCO, Scopus via Elsevier, Wiley/Web of Science and Cochrane Library. The selected databases were chosen according to the health care setting and the concept of employee voice. Therefore, health-related databases and business-related databases were used. A three-stage search strategy guided by the manual for literature search in specialized databases, RefHunter [35], was used. Specifically, the three phases include a preliminary search, a systematic search, and a supporting search. As previously described, a preliminary search of MEDLINE via PubMed was performed to refine the inclusion and exclusion criteria, to define the search components, and to identify key words and MeSH terms. The preliminary search was conducted by KA and discussed by the research team. As a result of the preliminary search, it became necessary to modify the selected inclusion and exclusion criteria, and consequently the search terms, in order to ensure the optimal scope and precision of the subsequent search. Specifically, the search was restricted to nurses, employee voice and digital tools, but no relevant results were yielded. Consequently, the scope was broadened to include healthcare organizations and, in addition, and all opportunities which address employee voice, not only digital tools. In the first step of the second stage, the systematic search, a search string in Medline via PubMed was developed by KA and discussed and adapted by the research team. The search string was then adapted for the other databases and the systematic search was conducted. Furthermore, in the third stage, we used citation tracking by screening the reference lists of relevant results and identified them via Google Scholar. The used search components, search terms and MeSH terms can be seen in the Table 3. Furthermore, the search terms and their synonyms are linked to search strings using the boolean operators (OR, AND). Filters were applied to the English and German languages. Truncations and MeSH terms were adapted to the databases used.

**Table 3:** Search strategy

Search components	Searchstring
**health care organization**	Health care organization or nursing area or care facilities or nursing institution or nursing care or long-term care [MESH] or specialties, nursing [mesh] or health facilities/nursing [MESH] or nurses [MESH]
	And
**opportunities**	Opportunities or instrument or intervention or tool or feedback tool or feedback instrument or digital feedback tool or digital feedback or evaluatoin instrument or digital voice channel or company mirror or channel
	And
**employee voice**	Employee voice or staff voice or speak* up or employee participation or employee involvement or work engagement (MESH)

### Study selection

**Fig. 1:**
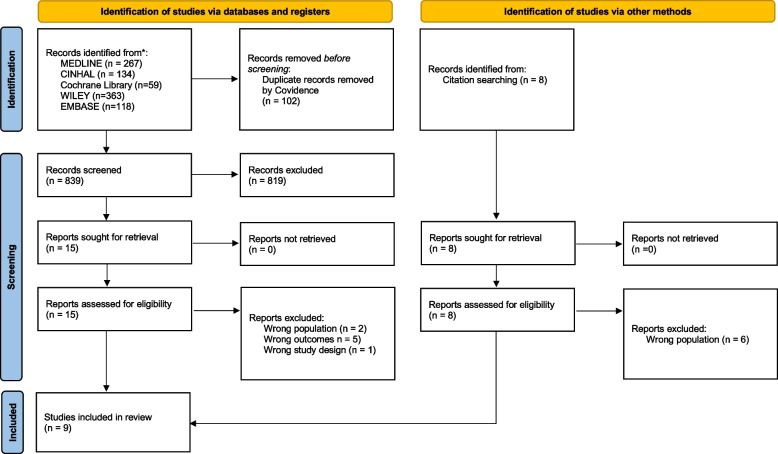
Prisma flow chart of the search

As shown in the PRISMA search flowchart (Fig. 1) we identified 951 studies in five databases and via citation tracking. All these results were imported into COVIDENCE. COVIDENCE is a software for managing and simplifying reviews. The software removed duplicates and allowed the two reviewers to screen the literature simultaneously. Specifically, the title and abstract were reviewed against the inclusion and exclusion criteria to identify appropriate studies separately by two researchers. The software identified disagreements between the two reviewers. Any disagreements were discussed in a follow-up meeting until a consensus was reached. If consensus could not be reached, a third reviewer (RM or FA) was involved. Once all open issues had been resolved, the full text analysis was carried out. The full text reports of the relevant studies were reviewed in the next step, just as in the abstract screening. The screening process was entirely carried out by two independent reviewers, following the inclusion and exclusion criteria (KA and BA). The studies selected by the two reviewers (KA and BA) were then discussed by the research team. The next step was to analyze the included studies.

### Data extraction

For the data extraction, an extraction form was developed based on TIDieR (Template for Intervention Description and Replication) [36] and CReDECI 2 (Criteria for Reporting the Development and Evaluation of Complex Interventions in health care) [37] by the two reviewers (KA and BA). The data extraction form included the following details: source (authors(s), year of publication, origin), aims, study design and methods, characteristics of the sample, outcomes and the definition of speaking up/employee voice, as well as a description of the opportunity to address employee voice. The data extraction form was developed by KA and validated by BA. To fulfill the search requirements, one reviewer (KA) extracted data, while the other reviewer (BA) cross-checked the data extraction. Disagreements between reviewers (KA and BA) were resolved by discussion. If consensus could not be reached, a third reviewer (RM or FA) was involved.

### Syntheses of extracted evidence

The included studies were heterogeneous in their definition and understanding of employee voice, described opportunities and contexts. Accordingly, the included studies are illustrated in Table 4 and presented in a narrative summary based on the aims of the review. This enables the identification of opportunities for employee voice and insights into the characteristics of these opportunities. The articles were analysed by the two reviewers (AK and BA). There was a regular exchange about the complete analysis [38].

**Table 4:** Characteristics of employee voice in health care

	**Definition of employee voice**			**Individual aspects of employees**	**Organizational aspects**	**Voice channels**
	**Employee voice**	**Speaking up (Safety climate)**	**Employee engagement**			
Adelman (2012) [39]	•				•	•
Clark et al. (2016) [40]			•		•	
Ginsburg and Bain (2017) [41]		•		•		
Kaine (2011) [42]	•			•	•	
Kee et al. (2021) [43]	•			•	•	
Krenz et al. (2020) [46]	•				•	
Law and Chan (2015) [44]		•		•	•	
Sexton et al. (2018) [45]	•				•	
Wilkinson et al. (2023) [5]	•				•	•

## Results

As seen in the PRISMA flow chart of the study selection and inclusion process in Fig. 1, we identified 951 studies in five databases and via citation tracking. After we removed 102 duplicates and screened 849 titles and abstracts, we assessed 23 full texts. All studies were published in English. From the 23 assessed studies, 8 were excluded because they did not include nurses, 5 were excluded because they did not address topics such as employee voice, speaking up or employee engagement. One study was a review and was therefore excluded. As a result, 9 studies were included in the review [5, 39–46]. The included studies were published between 2012 and 2023. Three studies were from the US [39, 40, 45], two from Australia [5, 42], one from Canada [41], one from the Netherlands [43], one from Hong Kong [44], and one from Switzerland [46]. Four reports used a quantitative study design [40, 41, 45, 46], and two used qualitative study design [43, 44]. A case study design was used in three studies [5, 39, 42].

It is important to note that the results of the scoping review do not provide evidence of the effectiveness of the interventions, as this was not the aim of the review.

The background to the drivers of the studies, such as why the interventions were implemented or researched, is usually not explicitly mentioned in the studies. Based on the description of the background and stated aims, seven studies were identified as focusing on patient safety [39–41, 44–46]. Three studies [5, 42, 43] state in their objectives that they want to better understand employee voice in health care. Consequently, the focus was placed on the organizational perspective. Kaine [42] aimed to explore employee voice in the aged care sector. The aim of Kee et al. [43] was to understand how members of lower status occupational groups can develop voice behaviors that transcend hierarchical levels. And the third study by Wilkinson et al. [5] analyzed the challenges of multicultural voice within the organization. The included studies [40, 45] report that there are valid evidence-based tools such as the *Healthy Work Environment Inventory [HWEI]* [40], the *Safety Attitudes Questionnaire* [47] or the *Safety, Communication, Operational Reliability & Engagement Survey* [*SCORE*™] [48] to assess different elements of a healthy work environment in the context of employee voice. The assessment instruments are not the primary focus of this review and therefore have not been subjected to detailed analysis. The results of the review are presented in Table 4. This table provides an overview of which definition of employee voice (speaking up, employee voice or employee engagement) was used in the included articles. Furthermore, we clustered the results according to the characteristics of the opportunities to address employee voice in categories such, individual aspects of employees, organizational factors, and voice channels. The included studies that address individual factors of employees to be able to raise their voice are summarized in the category of *individual aspects of employees*. The category of *organizational factors* is a description of the organization’s approach, such as the role of the management or the organizational culture. *Voice channels*, or opportunities through which employees can express their opinions, are included in the fourth category.

### Individual aspects to address employee voice

In addition to the aspects of organizational culture and the role of management that have been described thus far, the employees themselves also play an important role in determining whether or not employee voice is possible. The included studies describe different ways to support the individual aspects of employees being able to speak up and initiate change [5, 41, 43, 44].

Using a pretest–posttest control group design, Ginsberg and Bain [41] developed a role-playing simulation workshop to promote speaking up. The aim of the research was to evaluate the impact of the multifaceted intervention to encourage staff to speak up when they have patient care concerns and when faced with unprofessional behaviors from team members. The survey was performed before the workshop and three and four months after the workshop at a community hospital in Ontario, Canada. The control group was the intensive care unit, and the intervention group was the emergency department. The primary participants were nurses, although physicians and other allied health professionals were also involved. The multifaceted intervention is described with the following components. First, there was a role-playing simulation workshop (opening monologues, debriefing sessions and an exit survey). Second, there were the follow-up interventions and the initiative for department-led leadership (staff huddles, one-on-one meetings). In summary, the multifaceted approaches that have been described can improve employees’ perceptions and, as a consequence, the team working climate. A good team working climate can further improve speaking up. The results refer to being aware of different context and internal organizational factors, as already described [41]. In a qualitative study, Kee et al. [43] analyzed how members of lower-status occupational groups can develop voice behavior that transcends hierarchical levels in the Netherlands. The study included observations of 5 Dutch home-care organizations and interviews with 14 registered nurses (4 years education), 10 auxiliary nurses (3 years education) and 12 supervisors. 14 participants joined a trajectory and were interviewed twice, halfway through and at the end of the trajectory. The trajectory was developed from the Dutch Nurses Association and included 8 full-day plenary training sessions, individual assignments, moments of personal reflection and individual coaching sessions. The role of members of low-status occupational groups are placed at the center of the research in this study. In doing so, it addresses the role of a specific group of employees and how they can have a voice in an organization. The results show that it is important for low-status occupational groups to receive special training in how to speak and how to give feedback. More specifically, there is a need for support to develop voice behavior to exert upward influence in their organization and initiate change that actually benefits their work group [

43].

Law and Chan [44] extend the findings of Kee et al. [43] that it is necessary to teach speaking up and that a continuous learning process is necessary from the beginning to generate a safe and open speaking culture. They used a narrative inquiry approach to explore the experiences of newly qualified registered nurses in speaking up. Their aim was to understand the learning process by which newly graduated registered nurses learn to speak up. These findings demonstrate that it is necessary to consider that a learning process is formed by cultural aspects and generational differences. In addition, they analyzed data showing that people need more than one-time training and safety tools to develop speaking up skills and promote culture. Suggested ways to support the learning process of speaking up include being mentored by others, self-mentoring and reflecting on mistaken experiences to see new possibilities for the future. More specifically, nurses were trained to speak up through mentoring before, during and after each experience of speaking up. Along with recommending that speaking up be seen as an ongoing learning process, this process can help to create an open and safe organizational culture [44].

Wilkinson et al. [5] also focused on the role of the employee in speaking up in their research, similar to Kee et al. [43]. In particular, they examined the impact of cultural issues on the voice of the employee. For example, they investigated whether ethnicity has an impact on whether employees speak up or remain silent. Their results show that regarding the cultural aspect, top-down communication can be misinterpreted. In general, the art of communication varies from one culture to another. In conclusion, CALD factors such as cultural norms, literacy, language and limited space have to be considered to build an organizational culture of employee voice.

The findings of Ginsburg and Bain [41], Kee et al. [43], Law and Chan [44], and Wilkinson et al. [5] showed that if the opportunities that deal with employee voice are to be used and have an impact, it is necessary to improve the level of education, provide ongoing training on how to use the opportunities, perhaps offer technical support and consider cultural and generational aspects.

### Organizational opportunities to address employee voice

Kaine’s [42], Sexton et al. [45] and Wilkinson et al. [5] found that there are opportunities such as the support and facilitator role of leaders, for example, through *WalkRounds*™ [45], to address and enable employee voice as a leader. However, for the opportunities to be effective, frameworks need to be embedded in organizations. Kaine's [42] case study explored employee voice in three aged care facilities in Australia. They conducted interviews with nurses and managers occupying a range of roles across the facilities, and they analysed internal documents. The findings suggest that there are various opportunities, which are described in the three cases that try to improve employee voice, such as a continuous quality improvement process (CQI), section staff meetings, clinical care committees, employee surveys conducted by third parties every 18 months and feedback forms. It is not clearly described in the studies that the nurses are using the trade unions to have a voice or to get a voice and how they are using them. In summary, the factors or opportunities that have an impact on the regulation of voice cannot be ranked in order of importance. However, the results of the case study highlight four areas that need to be considered to address employee voice in aged care: location, social norms, labor law and organizational norms. These findings clearly indicate that the location of an organization (local labor market), social norms (social meaning of elderly care), the expectations of the labor market and labor law interact with the internal regulation of an organization. Such internal regulations are most often expressed through the exercise of the management. This leads to the fact that leaders have a key role in enabling employees to have a voice in the organization [42].

Wilkinson et al. [5] concluded in their single-case study residential aged care facilities in Australia. The aim of this research was to understand the challenges of multicultural voices within an organization (managerial and employee perspective) in terms of what encourages or inhibits the propensity of employees to speak up. The analysis of 21 semistructured interviews, 10 interviews with managers and 11 with hospitality and nursing staff in one aged care facility highlights a voice system that includes voice and communication channels, as well as the designers of the voice system. Furthermore, the identified voice system blockages. Blockage in the voice system is summarized in three factors: management (lack of safety, futility, no dobbing culture), organizational (lack of skills to use voice opportunities and budget) and CALD factors (cultural norms, literacy, language, limited space to build social capital).The analysis of the designers involved in the voice systems showed that the management genuinely cared about the communication and voice processes in the organization. However, the focus was more on ensuring that all staff were informed and less on giving upward feedback to initiate change [5].

*WalkRounds*™ (WR) is a tool for connecting leadership to patient safety and inculcating safety ideas into health care systems. The WR consists of a core group (employees such as nurses and other available staff), which includes executives who conduct weekly visits to different areas of the hospital. The aim is to talk about specific questions, adverse events and the factors or system issues that led to these events. In summary, it is an informal opportunity for leaders to have discussions about safety issues with front-line staff [49]. The findings of Sexton et al. [45] provide important insights into the effects of WR. This cross-sectional web-based survey of 31 hospitals used the SCORE to understand relationships between WR with feedback and health care work setting norms (safety culture, employee engagement, burnout and work-life balance). The sample consisted of a diverse range of healthcare professionals and administrative staff. Of these, nurses constituted the largest proportion of any professional group, representing approximately 31% of the total sample. The results show that work settings reporting more WR with feedback had substantially higher safety culture domain scores and significantly higher engagement scores for four of its six domains. In conclusion, the authors recommended that WR with feedback is a potential intervention to address speaking up [45]. In a case study of four hospitals, Adelman [39] conducted a document analysis and interviewed individuals in various roles within each hospital, including the Chief Executive Officer and frontline nurses. The results of this study represent key elements of leadership promoting employee voice. Each of their key elements – culture, voice opportunity, risks and costs, voice instrumentality – positively influenced employee voice. Voice opportunities include investigations to give employees the opportunity to speak up through both formal and informal communication channels. For these opportunities to be used and work, a certain leadership behavior is needed. The results demonstrated that approachability and visibility are important. Leaders need to be present and visible in their organization. They are role models and should offer face-to-face communication and listening. *Voice instrumentality* refers to leaders’ response and impact when employees speak up. What happens when employees give feedback? What consequences and responses might follow? The key elements of *culture*, risk and costs describe the organizational culture. This includes whether employees felt comfortable talking about their concerns. In practical detail, this involves psychological safety, relationships, and trust as important factors when employees speak out or remain silent. It also includes education and training to learn to speak up to further create continuous improvement [39].

Krenz et al. [46] showed that the formal hierarchy in a team as well as the behavior of the team leader can influence nurses’ voice. They analysed 78 different health care providers (36 nurses, 29 junior doctors, 13 consultants) in a large university hospital during ten different one-day simulation-based team training sessions. The results provide a clear account of the effects of leadership and hierarchy on nurses' actual voice. They show that hierarchy and leadership delay nurses' first voice but do not affect the overall frequency of their voice [46].

### Voice channels

In their research, Adelman [39] describes voice opportunities as key element of promoting employee voice. As already mentioned, voice opportunities should contain formal and informal communication channels. Formal communications approaches that were used in the four analyzed cases were, for example, rounding logs to track trends, tracking employee recognition, and following up on issues. All CEOs discussed rounding as their primary means for soliciting upward feedback. Additionally, computerized communication and feedback repositories for employees to submit questions or concerns directly to senior leaders were mentioned. Further formal approaches to communication were ad hoc focus groups, compliance hotlines, cross-functional committees, meetings, retreats, employee culture and opinion surveys, knowledge boards, suggestion boxes, volunteer satisfaction surveys, steering committees, process improvement committees, and one-on-one meetings/luncheons. The informal communication strategies used in the four cases were e-mail or telephone calls and an open-door policy. Furthermore, they mentioned BBQ days, employee breakfasts and luncheons as informal ways to communicate. One case also described the use of podcasts, blogging, and other social media to communicate in the variety of ways that people may prefer [39].

In their case study, Wilkinson et al. [5] describe a *voice system* that includes voice and communication channels. The voice and communication channels used in the analyzed long-term care facilities were divided into upward (top down) and downward (bottom up) channels that carried information to or from employees. In the explored case, the bottom-up tools that were implemented included learning circles, open door policy, improvement log, whistleblowing line and informal voice, performance reviews and climate surveys. To communicate information to employees (top-down), Wilkinson et al. [5] identified and described the use of a touchscreen tablet messaging system, communication rooms, newsletters/magazine and access to information via an intranet system. Furthermore, the findings indicate that communication systems that are oriented top-down management ought to be considered in order to prevent misinterpretations of diverse work cultures. Additionally, the long-term care facilities used opportunities for two-way communication, such as different forms of meetings, a communication system within the company and daily briefings/shift handovers [5].

## Discussion

The characteristics of the nurse employee voice opportunities described in the included studies were summarized. Three main characteristics that need to be considered to enable nurses to have a voice in the organization were identified. These main characteristics are individual factors, organizational culture, and available voice channels. It is not possible to rank them in order of importance; they are interrelated.

The individual factors include the knowledge and skills that a nurse needs to be able to give voice, as well as internal and external motivation. One of the most mentioned opportunities for individual factors was the education of the nurses [18, 41, 43, 44]. It is an ongoing education to learn how to raise your voice, which starts in basic education and needs to be trained through mentoring in daily work [41, 43, 44]. Therefore, the education level has an influence on whether employees are able to communicate feedback and take the opportunity to speak up and use supportive structures in a way that change is initiated and not in the way of complaints [43].

Lee et al. (24) summarize that individual factors that influence speaking up are internal motivation toward patient safety, perceived effectiveness and importance, assertiveness, and organizational commitment. The ability to be assertive is an individual factor that can be trained. The factors of perceived effectiveness and importance from employees are described in this review in the organizational culture because perceived effectiveness and importance from employees need to be addressed by management.. Kaine [42] argues that the location of an organization (whether there other job opportunities for employees) and the social norms (how care is generally perceived) influence voice and silence. Wilkinson et al. [5] add that cultural norms, literacy, language, and limited space to build social capital can mute employee voice. Lee et al. [24] discussed similar findings in the sociocultural context and showed that Asian sociocultural characteristics (age and seniority hierarchy, collectivist culture, and professional and gender differences) made nurses reluctant to speak up. This leads to the perception that sociocultural characteristics have an impact on whether nurses speak out or remain silent. Overall, the findings of this review show that employees need to learn how to have a voice in their organization through mentoring or training to raise their concerns and then try to influence their work and the performance of their organization through their voice [5, 13, 18]. In addition, employee voice should be seen as an ongoing learning process in the organization, considering individual, social and cultural aspects. Consequently, to be effective, these individual factors require an appropriate organizational culture.

Organizational culture includes all investigations in organizations and opportunities that should be considered to support employee voice in an organization. According to Adelman [39], an organization’s mission, values and vision guide its culture. Therefore, to achieve the organizational commitment and employee perceived efficacy and importance described by Lee et al. [24], these values and visions must be implemented and become routine.

In creating a culture where nurses can have a voice, the management of the organization can be the designers of voice systems and role models [5, 46]. Management determines employee voice by deciding which voice channels are used or not used. In addition, external and internal regulation (organizational and labor law) are multifaceted. In different circumstances, different aspects of the same regulatory constraint will be more important than others [42]. More specifically, management should be visible and easy to contact [39], support structures such as WalkRounds [45], and offer face-to-face communication [39] or opportunities to improve the relationships among lower and higher status employees, which allow nurses to have a voice and support a safe work environment [43]. Morrow et al. [16] and Lee et al. [24] support these findings and emphasize the role of management. The meta-synthesis by Morrow et al. [16] of 11 qualitative studies shows that hierarchies and power dynamics negatively affect safety voice and embedded expectations of nurse behavior. Additionally, they summarized that nurse managers have a powerful positive or negative affect on safety voice [16]. Umoren et al. [18] add that the impact of hierarchical structures on employee voice can be modified by addressing institutional, interpersonal, and individual factors.

Lee et al. [24], like Krenz et al. [46], added team factors to be considered in a healthy, open, and safe work environment. These include positive relationships and team trust, team culture and mentoring. Overall, to support nurses’ willingness to submit voice, the organizational culture must be safe and open, and sociocultural characteristics and work positions need to be considered [5, 24, 43]. Therefore, open and supportive managers play a vital role [24]. Umoren et al. [18] confirm this statement and recommend empowering managers to actively encourage and reward employee voice. In addition, the studies do not clearly describe whether participatory codetermination is an outcome of employee voice. However, the content may indicate that employee voice is a part of participatory codetermination. Furthermore, from the synthesis of the studies it can be argued that trust is an essential aspect that enables employee voice, and at the same time employee voice can also create trust in the organization [19, 20].

In summary, nurses need to know how to raise their voice, and they need to have a safe, open and learning organizational culture that allows them to have a voice in their organization.

Nurses also need to have opportunities, such as voice channels, where they can make their voices heard. The opportunities mentioned in the results are supportive structures and communication opportunities. The findings demonstrate that formal and informal opportunities for communication are ways to offer possibilities to raise voice [39]. Furthermore, Wilkinson et al. [5] describe top-down and bottom-up communication channels. It should be noted that top-down information can also be misunderstood due to language barriers or cultural differences, so it is important to take these into account and offer both top-down and bottom-up options.

Taken together, the current situation can be analyzed and assessed, and ways of improving employee voice can be identified and changed using assessment tools that measure employee engagement, safety/work culture or individual attitudes. The described possibilities, are not effective on their own. For all these opportunities, it is necessary to consider the working environment (social-norms, cultural, gender and generational aspects), the continuous learning process and the training of each individual employee as well as managers. As already Jones et al. [30] summarized there is no one-size-fits-all approach. In addition to these influencing factors and contextual conditions, it is important to be aware that the nature, focus, context, and severity of the problem affects the individual situation of speaking up or remaining silent [2].

It should also be noted that the studies were conducted from the perspective of different disciplines, namely from the perspective of human resource management and from the perspective of organizational change, which leads to a combination of two approaches. On the one hand, the focus is on the employees in the company and, on the other hand, on the organizational changes. Given the definition of employee voice, that employees use their voice to challenge processes and initiate change [15, 16], and the described characteristics of the opportunities to address employee voice, it is worth discussing these two approaches. A separation of these approaches does not seem to make sense, and the approaches influence each other. On the one hand, employee empowerment and retention, culture of trust, individual aspects, and on the other hand, the constantly changing health care system and organizational culture.

## Limitations

The definition of employee voice in the literature is inconsistent; therefore, we decided to include all studies that address employee voice, work engagement, or speaking up, with a specific focus on opportunities that address giving nurses a voice in health care providers.

The small number of included studies is due to the fact that only few focus specifically on nurses. Since this review is part of a larger research project on the promotion of the voice of nurses, our narrow focus was necessary. Further information can be found in the research protocol [50]. It must be considered that speaking up, as mentioned above, is often related to safe work environments and does not include employee voice. However, speaking up is a necessary part of employee voice, so these findings can also be seen as relevant to address employee voice. This is why the research team decided to explicitly describe the definition of employee voice that is used in the included studies when extracting the data. Considering the inconsistent definitions of employee voice in the studies, it is also difficult to summarize what exactly the studies were trying to achieve in terms of speaking up/having a voice or reducing silence. As described in the background, the two concepts of silence and voice need to be considered separately when implementing opportunities. It should also be noted that the used voice channels were not sufficiently described in the studies included.

## Conclusion

To conclude, employee voice is a process. In order for utilize employee voice opportunities, individual employee factors, organizational culture and its embedded context must be considered. This requires staff empowerment, so they voice their concerns and managers to encourage and act on this feedback. A safe, open, and trusting work environment is critical because it fosters continuous learning and promotes constructive communication. Individual internal and external motivation, which is influenced by socio-cultural aspects and work hierarchies, must also be considered for successful use of opportunities.

## Supplementary Information

Supplementary Material 1.

## Data Availability

The data that support the findings of this study are available in the supplementary material of this article. Further details are available from the corresponding author upon reasonable request.

## Abbreviations

ADVICE: UnderstAnDing Employee VoICe Behavior (acronym for the study)
HWEI: Healthy Work Environment Inventory
SCORE™: Safety, Communication, Operational Reliability & Engagement Survey
