# Supplementary material for: Opportunities for nurses to address employee voice in health care providers: a scoping review
Source: BMC Nurs. 2024 Sep 13;23:651. doi: 10.1186/s12912-024-02331-y (PMC11401326; doi:10.1186/s12912-024-02331-y)
Supplement: Supplementary file 1 — Supplementary Material 1. [file 12912_2024_2331_MOESM1_ESM.pdf]

Supplementary Table S1: Details of included studies

Source	Aim	Design/Methods	Sample	Definition employee voice	Opportunity to address employee voice
Adelman (2012) [39]<br><br>USA	Understand CEO behaviors and actions that promote employee voice and upward communication in health care organizations. Further to understand how CEO's foster employee voice and upward communication of both positive and negative information in their organization?	Phenomenological collective or multiple, case study approach.<br><br>Data Collection:<br>• document review<br>• 20 semi-structured telephone interviews<br><br>Data analysis:<br>phenomenological reduction organizes textural meanings and patterns into themes<br><br>ATLAS.ti 6 analytical software	two national-level MBNQA[1] and two state-level performance excellence award–winning hospitals<br><br>Case 1: 14.000 employees, not-for-profit regional system, CEO tenure 4 years<br><br>Case 2: 4.000 employees, locally owned, not-for-profit regional hub, CEO tenure 7 years<br><br>Case 3: 2.080 employees, governmental, not-for-profit community hospital, CEO tenure 5,5 years<br><br>Case 4: 5.200 employees, not-for-profit regional system, CEO tenure 4 years<br><br>[1] Malcolm Baldrige National Quality Award® is the highest level of national recognition for performance excellence that a U.S. organization can receive (NIST, 2023)	Employee voice is defined in this study after Detert and Burris (2007)[2] as the discretionary provision of information intended to improve organizational functioning to someone with the authority to act.<br><br>[2] Detert, J. R., and E. R. Burris. Leadership Behavior and Employee Voice: Is the Door Really Open? Academy of Management Journal. 2007. 50 (4): 869–84.	Visibility and Approachability<br>CEO's were present and visible in their organizations.<br><br>Culture<br>Focus on the organization's culture (continuous improvement guided by the organization's mission, vision, values, and established employee behavior standards).<br><br>Formal Communication Strategies<br>focus groups, hotlines, cross-functional committees, department/unit orientation, meetings, retreats, electronic performance scorecards, employee-leader forums, employee culture and opinion surveys, employee luncheons, employee recognition celebrations, newsletter, website, boards, education, learn and lead programs, monthly CEO report, new employee orientation, one-on-one meetings, process improvement teams, reporting hierarchy, rounding logs, rounding, computerized communication and feedback, steering committees,<br><br>Informal Communication Strategies<br>e-mail or telephone calls, open-door policies, meetings, BBQ days, employee breakfasts and luncheons, podcasts, blogging, and other social media
Source	Aim	Design/Methods	Sample	Definition employee voice	Opportunity to address employee voice
Clark et al. (2016) [40]<br><br>USA	Measure nurses' and nursing faculty's perceptions of the health of their work environment and describe the development and psychometric testing of the Healthy Work Environment Inventory (HWEI).	Exploratory factor analysis of the HWEI<br><br>Designing the HWEI:<br>based on experience and expertise, literature review<br>20-item Likert-type survey<br>20 essential elements of a healthy work environment<br><br>Data analysis:<br>Kaiser-Meyer-Olki 0.50 or greater<br>Bartlett's test p<.05<br>Promax<br><br>Data collection:<br>Web-based and paper-and-pencil version during four nursing workshops	520 nurses<br>• nursing faculty<br>• practice-based nurses throughout the USA	According to the American Nurses Association (ANA, 2016)[3], a healthy work environment is one that is safe, empowering, and satisfying, and where all leaders, managers, health care workers, and ancillary staff perform with a sense of professionalism, accountability, transparency, involvement, efficiency, and effectiveness while being mindful of the health and safety of all individuals.<br><br>[3] American Nurses Association. (2016). Healthy work environment. Retrieved from: http://www.nursingworld.org/MainMenuCategories/WorkplaceSafety/Healthy-Work-Environment	The HWEI is an evidence-based tool to assess the elements of a healthy work environment, raise awareness, and determine strengths and areas for improvement. Step toward improving the workplace and promoting individual, team, and organizational success.<br><br>The HWEI is a basis for interview questions.<br><br>All interitem correlations were positive and statistically significant. The HWEI appears to be a 1-factor scale that measures the same underlying construct of a healthy work environment. The scale is both reliable and internally consistent based on Cronbach's alpha (>0,70).

Source	Aim	Design/Methods	Sample	Definition employee voice	Opportunity to address employee voice
Ginsburg and Bain (2017) [41]<br><br>Canada	Develop a role-playing simulation workshop designed previously to promote speaking up behaviors.<br><br>Evaluate the impact of a multifaceted intervention on the teamwork climate to improve communication and encourage staff to speak up when they have patient care concerns or when faced with unprofessional behaviors from team members.	Pretest-posttest control group design<br><br>Teamwork Climate Survey, educational outcomes and Exit survey level 1<br><br>Timing of the surveys:<br>T1 = before the workshop (June 2014)<br>T2 = 3 months after the workshops, shortly after completion of the weekly discussion briefings<br>T3 = 4 months after (January 2015)<br><br><u>Data analysis:</u><br>ANOVA<br>Fisher's Exact test<br>Wilcoxon	Southlake Regional Health Centre (SRHC) a large community Hospital, Ontario, Canada<br><br>Control Group – ICU[4]<br>Intervention Group – ED[5]<br><br>42 workshop participants and survey participants for T1, T2 and T3 were 54% nurses, 2,6% physicians, 20,5% health care professionels from the ICU and the ED<br><br>T1: ED 83, ICU 29<br>T2: ED 39, ICU 23<br>T3: ED 38, ICU 14<br><br>[4]Intensive Care Unit<br>[5]Emergency Department	Accountability Framework at Southlake Regional Health Center (SRHC) – tool kit will provide staff with phraseology and self-management techniques to be applied in situations where clinical issues and/or professional behaviors arise.	**Multi-faceted intervention (three components):**<br>1) <u>Role-playing simulation workshop</u><br>opening monologues, introduction of the SRHC, use of these communication tools from the SRHC in role playing, debriefing sessions, and an exit survey.<br><br>2) <u>Follow-up interventions</u><br>six weekly discussion briefings were held with interprofessional staff during the two-month period that followed; each discussion was summarized and shared in a weekly ED meeting.<br><br>3) <u>Department-led leadership initiative</u><br>commitment from the ED leadership Following (T1), 10-minute staff huddles were instituted three to four times daily, where ED staff were encouraged to provide input on any aspect of ED operations. One-to-one meetings with every staff member were held to review performance and obtain feedback.<br><br>**Outcome:**<br>A multifaceted approach to improving communication can improve employees' perceptions and the team's working climate. Changing communication behaviors and creating a climate that supports speaking up is challenging. Continued efforts to intervene and improve are needed, context-dependently.
Source	Aim	Design/Methods	Sample	Definition employee voice	Opportunity to address employee voice
Kaine (2011)[42]<br><br>Australia	Exploring employee voice in the aged care sector using insights offered by a regulatory approach.<br><br>Understand and consider voice in new and different ways.	qualitative case study methodology<br><br>3 cases: three aged care facilities in New South Wales<br><br><u>Data collection:</u><br>- internal documents (employment policies, strategic plans, newsletter, websites, minutes of meetings, annual reports, histories)<br>- 20 in-depth interviews	3 aged care facilities are from different distinct parts of the sector.<br><br>1) community-run, not-for-profit<br>2) not-for-profit, religious, charitable<br>3) run for-profit<br><br><u>Interview participants:</u><br>personal care assistants,<br>enrolled nurses,<br>registered nurses,<br>care services employees,<br>administration and HR managers,<br>health service manager,<br>hostel manager,<br>CEO and chairman of the board.	**Implemented Voice Mechanism:**<br>Case 1: continuous quality improvement processes (CQI), OH&S committee, section staff meetings, clinical care committees<br><br>Case 2: employee survey conducted by a third party every 18 months; feedback forms; OH&S committee; staff meetings; clinical care committees<br><br>Case 3:<br>General staff meetings; suggestions and complaints forms; clinical care committees; OH&S committee<br><br>Little countervailing regulatory pressure being imposed by workers in the form of unions or other methods of voice.	The factors cannot be ranked, so the results show that each organization needs to create its own overlapping regulatory web that manifests differently.<br>Managing employee voice requires recognizing not only the regulatory factors themselves but also that the factors are interrelated and subject to change.<br><br>**Regulation and voice:**<br><u>Location:</u> importance of location and local labor market conditions on how particular organizations and employees experience voice and turnover (alternative employment options available)<br><br><u>Social norms:</u> include the social meaning of care; work determines how care workers interact with the labor market, what they expect from their jobs, and therefore, potentially, their willingness to speak up.<br><br><u>Labor law:</u> managers decide whether or not workers have avoice, and it is managers rather than employees who decide what mechanisms to utilize.<br><br><u>Organizational norms:</u> external and internal regulation are multi-faceted, and in different circumstances, different aspects of the same regulatory constraint will be more important than others.

Source	Aim	Design/Methods	Sample	Definition employee voice	Opportunity to address employee voice
Kee et al. (2021) [43]<br><br>Netherlands	Understand how members of lower-status occupational groups can develop voice behavior that transcends hierarchical levels.<br><br>Show how the development of voice behavior allows subordinates to exert upward influence in their organizations and initiate change that actually benefits their own occupational group.	Interpretative research approach<br><br><u>Data Collection:</u><br>36 semi-structured interviews in two points time (halfway through and at the end of a trajectory)<br><br>Observational data from meetings, training sessions<br><br>Reflection reports from the participants at the trajectory<br><br>Trajectory (develop from the Dutch Nurses Association)<br>8 full day plenary training sessions, individual assignments, moments of personal reflection and individual coaching sessions	Dutch home-care organizations<br><br>14 participants at the trajectory<br><br>Interviews with the participants, participants colleagues and supervisors.<br><br>12 supervisors,<br>14 registered nurses (4 years education)<br>10 auxiliary nurses (3 years education)	Regarding to Van Dyne and LePine (1998) Kee et al. (2021) define voice as a more constructive type of behavior through which employees can positively impact or change the functioning of an organization.	Members of low-status occupational groups can develop voice behavior that transcends hierarchical levels; the development of knowledge as well as relationships between different occupational groups are crucial for this.<br><br>**Development knowledge:**<br>Special training in how to speak and how to give feedback.<br>How the development of such voice behavior allows subordinates to exert upward influence in their organizations and initiate change that actually benefits their own occupational group.<br><br>**Additional Opportunities:**<br>• Organizations share policy documents with low-status employees.<br>• Facilitate employees development of knowledge about how to communicate and interact.<br>• Implement a support structure (to experiment and give each other feedback).<br>• Implement visits from mangers at the work floor or implement a workday with a manager.
Source	Aim	Design/Methods	Sample	Definition employee voice	Opportunity to address employee voice
Krenz et al. (2020) [46]<br><br>Switzerland	They want to understand how changes in hierarchy and leadership are associated with nurse voice frequency and nurses time to speak up during simulated acute care situations.	Quantitative study<br><br><u>Data Collection:</u><br>video recordings from training sessions (one day simulation-based training)<br><br><u>Data analysis:</u><br>INTERACT coding software	University hospital<br><br>10 training days with total of 78 participants<br><br>36 nurses<br>29 residents<br>13 consultants<br><br><u>Age:</u><br>24-29 (11 participants)<br>30-35 (23 participants)<br>36-41 (20 participants)<br>42-57 (9 participants)<br>48-53 (7 participants)<br>>53 (6 participants)<br><br><u>Work experience (years):</u><br>>2 (2 participants)<br>3-4 (10 participants)<br>5-6 (9 participants)<br>7-8 (10 participants)<br>9-10 (4 participants)<br>>10 (41 participants)	They chose to use the term *nurses' voice*, which refers to utterances by nurses involving either suggestion-, problem-, opinion, or doubt-focused content.	Hierarchy as well as leadership delay nurses' first voice but do not affect overall nurses' voice frequency.<br><br>Formal hierarchy in a team as well as team leaders' behavior can affect nurses' voices.

Source	Aim	Design/Methods	Sample	Definition employee voice	Opportunity to address employee voice
Law and Chan (2015) [44]<br><br>Hong Kong	The aim of this study was to explore the process by which NGRN's[6] learn to speak up in practice.<br><br>[6]Newly graduated registered nurses [NGRN's]	Narrative inquiry, Storytelling<br><br>Data Collection:<br>• individual unstructured interviews<br>• ongoing mails with 3 participants (monthly, share their experiences),<br>• document analysis (reviewing relevant hospital documents relating to their stories)<br>• focus group interviews<br><br>Data Analysis:<br>Iterative, on two levels regarding to Strauss and Corbin (1990)	Public Hospitals of Hong Kong Hospital Authority<br><br>Interview participants:<br>18 NGRN's 12,18, and 24 months after registration<br><br>3 stories from 3 NGRN's (neurosciences, neonatal intensive care unit and special baby care unit) were used for the analysis and the ongoing mail conversation.<br><br>Focus group participants:<br>- four trainer groups<br>- stakeholder group (senior nurses, ward nurses, physician)	Hong Kong Hospital Authority, speaking up program based on Crew Resource Management [CRM]<br><br>1-day interdisciplinary classroom-based CRM program teaching about CRM concepts and three safety tools (MEWS, ISBAR, Assertion model) for nurses, physicians, and other health care professionals<br><br>MEWS = modified early warning score<br><br>ISBAR = standardized team communication approach<br><br>Assertion model = communication model (1. Get persons attention, 2. Express concern, 3. State problem clearly and concisely, 4. Propose action, 5. Reach decision)	**Learning to speak up requires more than one-off training and safety tools:**<br>• ongoing education and experiences in building confidence<br>• training in speaking up; mentoring before, during, and after each experience of speaking up<br>• peer-mentoring and self-mentoring (explanations when voices are ignored)<br>• making public spaces safe for telling secret stories<br>• cultivation of a culture of safety (open and safe culture of communication)
Source	Aim	Design/Methods	Sample	Definition employee voice	Opportunity to address employee voice
Sexton et al. (2018) [45]<br><br>USA	Understand relationships between WalkRounds™ (WR) with feedback and health care work setting norms (safety culture, employee engagement, burnout, and work-life balance).<br><br>WR: front-line health care workers are encouraged by leadership to identify and resolve issues related to the safe delivery of care (a form of observable leadership engagement).	Cross-sectional web-based survey of 31 hospitals<br><br>WR analysis with the SCORE<br><br>2-month period 2015<br><br>Data analysis:<br>RE-ANOVA's and ICC's (Analysis of variance and interclass correlation)	16 797 participants (health care worker)<br>839 work settings<br>31 hospitals in Michigan, USA<br><br>Participants:<br>nurse (4548)<br>nurse aide (672)<br>other health care worker (2918)<br>clinical social worker, clinical support (medical assistant), pharmacist, dietitian, physician, technician, therapist, clinic manager, supervisor, administrative support<br><br>Years in specialty:<br>6 months or less (4%)<br>5-10 years (21,7%)<br>11-20 years (23,8%)<br>21 years or more (20,7%)<br><br>16% academic teaching hospitals<br>55% 99 or fewer licensed beds<br>16% 100-199 beds<br>19% 200-299 beds<br>12% over 300	SCORE (Safety, Communication, Operational Reliability, and Engagement) is a valid survey that measures the consensus view of workplace norms within a group of people.<br><br>Main topics: improvement readiness (learning environment), local leadership, burnout climate and personal burnout, teamwork climate, safety climate	32,7% participate in WR<br>24,3% participate in WR with feedback<br><br>Work settings reporting more WR with feedback had substantially higher safety culture domain scores (first vs. fourth quartile Cohen's d range: 0.34–0.84; % increase range: 15–27) and significantly higher engagement scores for four of its six domains (first vs. fourth quartile Cohen's d range: 0.02–0.76; % increase range: 0.48–0.70)<br><br>New safety culture domains of improvement readiness and local leadership were substantially higher in work settings where WRs were conducted with feedback.

Source	Aim	Design/Methods	Sample	Definition employee voice	Opportunity to address employee voice
Wilkinson et al. (2023) [5]<br><br>Australia	Understanding the challenges of multicultural voice within the organization (managerial and employee perspective) in terms of what encourages or inhibits the propensity of employee voice.	Qualitative approach<br>single-case study<br><br>Data collection:<br>• primary semi-structured interviews<br>• newsletters and policies<br><br>Data analysis:<br>Content analysis by two research members independently	Residential aged care facilities CareCo located in a large capital city in Australia<br><br>145 beds<br>182 employees<br>154 females and 82 males, and over 53% of the employees were born in countries other than Australia<br><br>21 semi-structured interviews<br>- 3 senior managers<br>- 7 middle management staff<br>- 11 hospitality and assistants in nursing (AN) employees who were from a range of culturally and linguistically diverse (CALD) backgrounds	Inclusivity in employee voice:<br><br>Understand the barriers that a particular group faces in voicing its voice and consider the ways this can be recognized and thus minimized.<br><br>Rethink the conceptualization of voice in how it relates more broadly to enhancing voice inclusivity within organizations that have a diverse multicultural workforce.	**Voice and communication channels:**<br>Upward (bottom up): learning circles, open doors, committees, focus groups, improvement logs, whistleblowing lines, informal voices, staff meetings, an in-house communication system, and a communication room; daily briefings and shift handovers; performance reviews; climate surveys<br><br>Downward (top down): staff meetings, an in-house communication system, a communication room; daily briefings and shift handovers; newsletters and access to information via CareCo's intranet system; touchscreen tablet messaging system<br><br>Communications systems are top-down-oriented, and misinterpretations are possible. Bottom-up channels ended up being narrowed.<br><br>**Designers of voice systems**<br>Voice was seen very much within the context of day-to-day management and communication within the work group.<br><br>**Blockage in the voice system**<br>Management factors: lack of safety; fear of voicing; no dobbing culture; a sense of futility<br>Organizational factors: a lack of IT skills had implications for access to voice opportunities.<br>CALD factors: cultural norms, literacy, language, and limited space to build social capital

Legend	
1	Malcolm Baldrige National Quality Award® is the highest level of national recognition for performance excellence that a U.S. organization can receive (NIST, 2023)
2	Detert, J. R., and E. R. Burris. Leadership Behavior and Employee Voice: Is the Door Really Open? Academy of Management Journal. 2007. 50 (4): 869–84.
3	American Nurses Association. (2016). Healthy work environment. Retrieved from: http://www.nursingworld.org/MainMenuCategories/ WorkplaceSafety/Healthy-Work-Environment
4	Intensive Care Unit
5	Emergency Department
6	Newly graduated registered nurses [NGRN's]
7	Intrapersonal factors scale [IPFS]
8	Postgraduate year [PGY]
